# Peritumoral Artery Scoring System: a Novel Scoring System to Predict Renal Function Outcome after Laparoscopic Partial Nephrectomy

**DOI:** 10.1038/s41598-017-03135-8

**Published:** 2017-06-06

**Authors:** Ruiyun Zhang, Guangyu Wu, Jiwei Huang, Oumin Shi, Wen Kong, Yonghui Chen, Jianrong Xu, Wei Xue, Jin Zhang, Yiran Huang

**Affiliations:** 1 Department of Urology, Renji Hospital Affiliated to Shanghai Jiao Tong University School of Medicine, Shanghai, China; 2 Department of Radiology, Renji Hospital Affiliated to Shanghai Jiao Tong University School of Medicine, Shanghai, China; 3 Department of Epidemiology and Statistics, School of Public Health, Shanghai Jiao Tong University School of Medicine, Shanghai, China

## Abstract

The present study aimed to assess the impact of peritumoral artery characteristics on renal function outcome prediction using a novel Peritumoral Artery Scoring System based on computed tomography arteriography. Peritumoral artery characteristics and renal function were evaluated in 220 patients who underwent laparoscopic partial nephrectomy and then validate in 51 patients with split and total glomerular filtration rate (GFR). In particular, peritumoral artery classification and diameter were measured to assign arteries into low, moderate, and high Peritumoral Artery Scoring System risk categories. Univariable and multivariable logistic regression analyses were then used to determine risk factors for major renal functional decline. The Peritumoral Artery Scoring System and four other nephrometry systems were compared using receiver operating characteristic curve analysis. The Peritumoral Artery Scoring System was significantly superior to the other systems for predicting postoperative renal function decline (p < 0.001). In receiver operating characteristic analysis, our category system was a superior independent predictor of estimated glomerular filtration rate (eGFR) decline (area-under-the-curve = 0.865, p < 0.001) and total GFR decline (area-under-the-curve = 0.796, p < 0.001), and split GFR decline (area-under-the-curve = 0.841, p < 0.001). Peritumoral artery characteristics were independent predictors of renal function outcome after laparoscopic partial nephrectomy.

## Introduction

Renal cell carcinoma (RCC) represents 2–3% of all cancers. Advances in radiologic techniques have facilitated the diagnosis of early-stage RCC, enabling partial nephrectomy (PN) as a treatment option. Laparoscopic PN (LPN) has been shown to have long-term oncologic and functional outcomes comparable to open PN^1–3^.

A negative surgical margin, no perioperative complications, and 90% renal function preservation comprise the trifecta outcome, which is used to determine an ideal PN outcome^4, 5^. Several nephrometry scoring systems predict surgical complexity and perioperative complication risk before surgery based on renal tumor anatomical complexity^6–8^. However, these systems have limited predictive value for postoperative renal function decline, which has a major impact on the ‘trifecta’ outcome^9, 10^. Numerous studies have demonstrated that tumor–renal anatomical characteristics affect postoperative renal function decline, but few have focused on the relationship between tumor–renal artery anatomy and outcome.

The primary aim of this study was to assess the impact of peritumoral artery characteristics on renal function outcomes, and to introduce our Peritumoral Artery Scoring System (PASS), which is based on three-dimensional (3D) computed tomography arteriography (CTA). We also examined the predictive value of PASS for renal function decline.

## Results

Clinicopathological patient characteristics and renal function outcomes are shown in Table 1 and Table 2. The median tumor size was 31 mm. The median RENAL^6^ and PADUA nephrometry scores were 8 and 9, respectively. The 12-month median postoperative estimated glomerular filtration rate (eGFR) percent decline was 7% (n = 182 patients [82.7%]). 77 patients (42.3%) suffered from a major eGFR decline (eGFR percent decline of ≥10% in total renal function, ePD10).

**Table 1:** Clinical variables and renal function outcomes of discovery cohort.

Characteristics	All patients n = 220	PASS-Low risk n = 98	PASS-Moderate risk n = 62	PASS-High risk n = 60	P-value
**Patient characteristics**					
Gender, no. (%)					
Male	159 (72)	70 (71)	45 (73)	44 (57)	0.981
Female	61 (28)	28 (29)	17 (27)	16 (43)	
Age, yr, median (IQR)	56 (47, 63)	54 (48, 63)	58 (47, 63)	54 (43, 65)	0.523
Body mass index, kg/m^2^, median (IQR)	23 (22, 25)	23 (22,26)	24 (23,25)	23 (22,25)	0.538
ASA score ≥ 2, no. (%)	61 (28)	29 (30)	17 (27)	15 (25)	0.823
**Tumor characteristics**					
Upper/Lower polarity, no. (%)	126 (57)	62 (63)	31 (50)	33 (55)	0.236
**Exophytic rate, no. (%)**					
≥50%	70 (32)	42 (43)	11 (18)	17 (28)	**<0.001**
<50%	117 (53)	49 (50)	38 (61)	30 (50)	
Endophytic	33 (15)	7 (7)	13 (21)	13 (22)	
Tumor size on computed tomography, mm, median (IQR)	31 (25, 43)	29 (22, 37)	34 (26, 42)	40 (27, 48)	**<0.001**
RENAL^6^ nephrometry score, median (IQR)	8 (7, 9)	7 (6, 8)	8 (7, 10)	8 (7, 9)	**0.001**
PADUA nephrometry score, median (IQR)	9 (7, 10)	8 (7, 9)	9 (8, 10)	9 (8, 10)	**0.002**
Resected and ischemic volume^13^ RAIV, cm^3^, median (IQR)	27 (17, 40)	20 (13, 28)	34 (20, 53)	37 (20, 59)	**<0.001**
**Operation characteristics**					
Operation time, min, median (IQR)	181 (123, 241)	177 (134, 234)	191 (124, 252)	173 (117, 242)	0.766
Warm ischemia time, min, median (IQR)	22 (20, 24)	21 (19, 23)	22 (20, 26)	23 (20, 25)	**0.009**
Estimated blood loss, ml, median (IQR)	210 (125, 280)	202 (108, 276)	205 (110, 280)	235 (155, 290)	0.235
Overall complications, no. (%)	23 (10)	8 (8)	6 (10)	9 (15)	0.372
**Pathological characteristics**					
Pathology staging, N (%)					
Benign	16 (7)	5 (5)	4 (6)	7 (12)	0.107
pT1a	190 (86)	90 (92)	54 (87)	46 (77)	
pT1b	14 (6)	3 (3)	4 (6)	7 (12)	
Surgical margin positive, N (%)	0 (0)	0 (0)	0 (0)	0 (0)	1.000
**Preoperative renal function base-line**					
eGFR, ml/min/1.73 m^2^, median (IQR)	107 (95, 123)	107 (97, 120)	103 (87, 117)	112 (89, 129)	0.108
**1-month renal function outcomes**					
eGFR absolute decline, ml/min/1.73 m^2^, median (IQR)	13 (2, 28)	5 (0, 18)	15 (7, 29)	22 (12, 32)	**<0.001**
eGFR percent decline, %, median (IQR)	13 (2, 24)	5 (0, 16)	15 (8, 29)	20 (12, 25)	**<0.001**
**12-month renal function outcomes**	**n = 182**	**n = 90**	**n = 47**	**n = 45**	
eGFR absolute decline, ml/min/1.73 m^2^, median (IQR)	7 (−2, 19)	1 (−6, 7)	10 (−2, 21)	19 (12, 25)	**<0.001**
eGFR percent decline, %, median (IQR)	7 (−2, 18)	1 (−6, 6)	9 (−2, 20)	18 (12, 23)	**<0.001**
eGFR percent decline ≥10%, no. (%)	77 (42)	13 (14)	23 (49)	41 (91)	**<0.001**

PASS = Peritumoral Artery Scoring System; IQR = interquartile range; ASA = American Society of Anesthesiologists.

**Table 2:** Clinical variables and renal function outcomes of validation cohort.

Characteristics	All patients n = 51	PASS-Low risk n = 24	PASS-Moderate risk n = 11	PASS-High risk n = 16	P-value
**Patient characteristics**					
Gender, no. (%)					
Male	33 (65)	18 (75)	9 (82)	6 (38)	**0.030**
Female	18 (35)	6 (25)	2 (18)	10 (63)	
Age, yr, median (IQR)	60 (48, 65)	53 (47, 63)	58 (53, 60)	61 (55, 68)	0.262
Body mass index, kg/m^2^, median (IQR)	23 (22, 25)	24 (23, 26)	24 (23, 25)	22 (21, 25)	0.127
ASA score ≥ 2, no. (%)	13 (25)	6 (25)	3 (27)	5 (31)	0.920
**Tumor characteristics**					
Upper/Lower polarity, no. (%)	26 (51)	12 (50)	6 (50)	8 (50)	1.000
Exophytic rate, no. (%)					
≥50%	15 (30)	8 (33)	1 (9)	6 (38)	0.165
<50%	26 (51)	14 (58)	6 (55)	6 (38)	
Endophytic	10 (20)	2 (8)	4 (36)	4 (25)	
Tumor size on computed tomography, mm, median (IQR)	28 (23, 43)	26 (20, 34)	37 (25, 41)	35 (25, 45)	**0.043**
RENAL nephrometry score, median (IQR)	8 (7, 9)	7 (6, 8)	8 (7, 9)	8 (6, 9)	0.154
PADUA nephrometry score, median (IQR)	9 (7,10)	8 (7, 10)	9 (8, 11)	9 (8, 10)	0.688
Resected and ischemic volume^13^ RAIV, cm^3^, median (IQR)	27 (15, 37)	18 (12, 25)	41 (26, 62)	27 (19, 39)	**0.010**
**Operation characteristics**					
Operation time, min, median (IQR)	190 (117, 243)	210 (159, 259)	150 (110, 180)	196 (112, 255)	0.312
Warm ischemia time, min, median (IQR)	23 (19, 25)	20 (18, 22)	23 (21, 29)	24 (20, 25)	**0.011**
Estimated blood loss, ml, median (IQR)	185 (110, 250)	195 (90, 258)	200 (108, 252)	175 (125, 228)	0.985
Overall complications, no. (%)	5 (10)	0 (0)	1 (1)	4 (25)	**0.025**
**Pathological characteristics**					
Pathology staging, N (%)					
Benign	6	1 (4)	1 (9)	4 (25)	0.214
pT1a	41	21 (88)	10 (91)	10 (63)	
pT1b	4	2 (8)	0 (0)	2 (13)	
Surgical margin positive, N (%)	1 (1)	1 (1)	0 (0)	0 (0)	1.000
**Preoperative renal scintigraphy base-line**					
Operated kidney GFR, ml/min/1.73 m^2^, median (IQR)	39 (34, 45)	40 (36, 46)	38 (36, 40)	37 (29, 45)	0.712
Total GFR, ml/min/1.73 m^2^, median (IQR)	80 (65, 90)	86 (75, 93)	75 (68, 78)	72 (57, 89)	0.382
**12-month postoperative renal scintigraphy**					
Operated kidney GFR absolute decline (oGAD), ml/min/1.73 m^2^, median (IQR)	9 (3, 15)	4 (1, 8)	14 (10, 15)	14 (9, 25)	**<0.001**
Operated kidney GFR percent decline (oGPD), %, median (IQR)	24 (10, 37)	10 (2, 18)	36 (27, 39)	37 (26, 39)	**< 0.001**
Operated kidney GFR percent decline of ≥20% (oGPD20), N (%)	29 (57)	5 (21)	10 (91)	14 (88)	**<0.001**
Total GFR absolute decline (tGAD), ml/min/1.73 m^2^, median (IQR)	8 (1, 17)	1 (−4, 9)	9 (3, 14)	11 (7, 29)	**0.001**
Total GFR percent decline (tGPD), %, median (IQR)	11 (1, 19)	2 (−5, 11)	14 (5, 19)	17 (11, 39)	**<0.001**
Total GFR percent decline of ≥10% (tGPD10), N (%)	27 (53)	6 (25)	7 (64)	14 (88)	**<0.001**

PASS = Peritumoral Artery Scoring System; IQR = interquartile range; ASA = American Society of Anesthesiologists.

**Figure 1:**
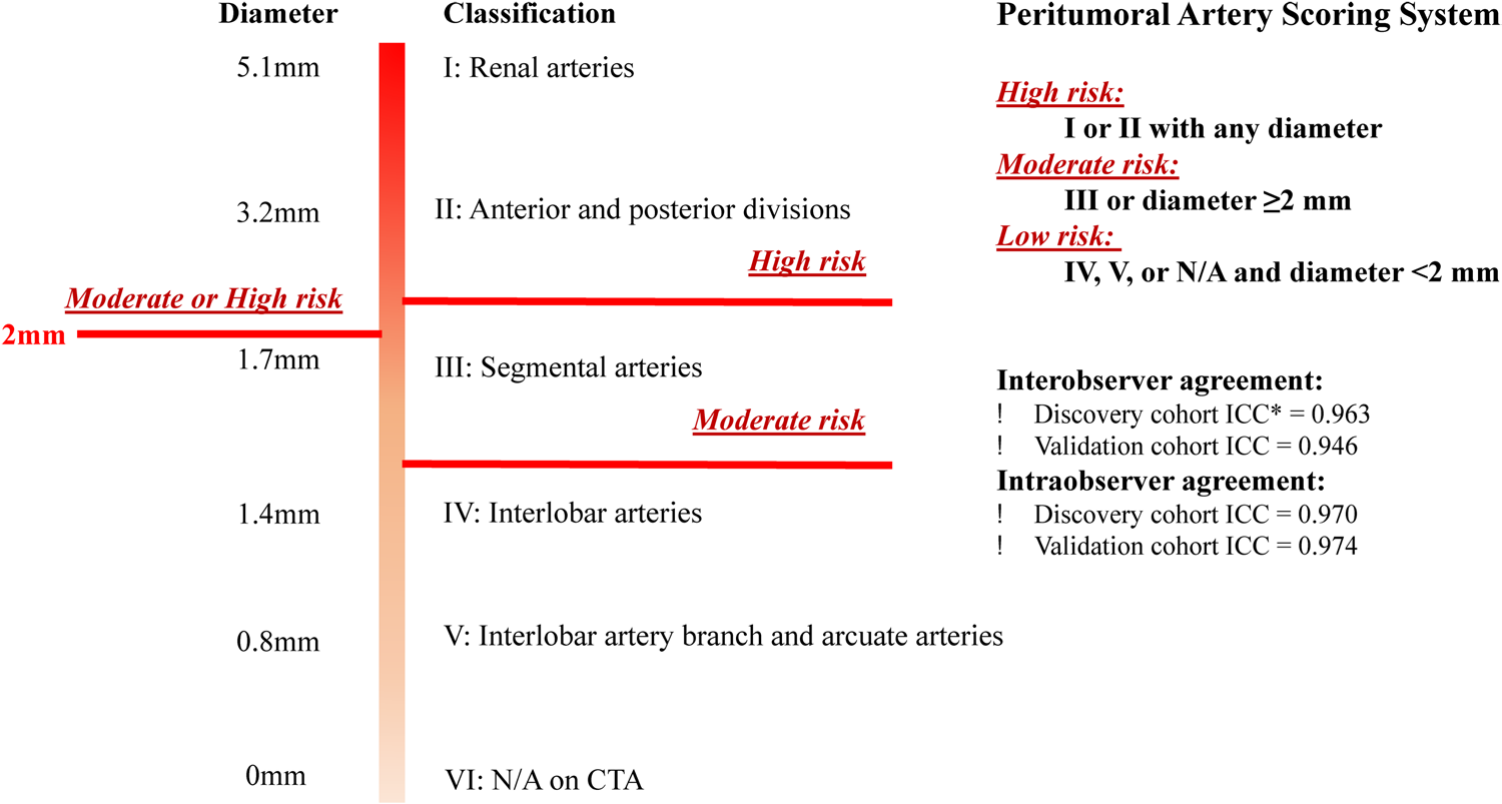
Spectrum of peritumoral arteries and definition of Peritumoral Artery Scoring System. The range of diameter was evaluated by 3D Volume Rendering (VR). *Interobserver agreements were assessed by using Intraclass Correlation Coefficients (ICC).

**Figure 2:**
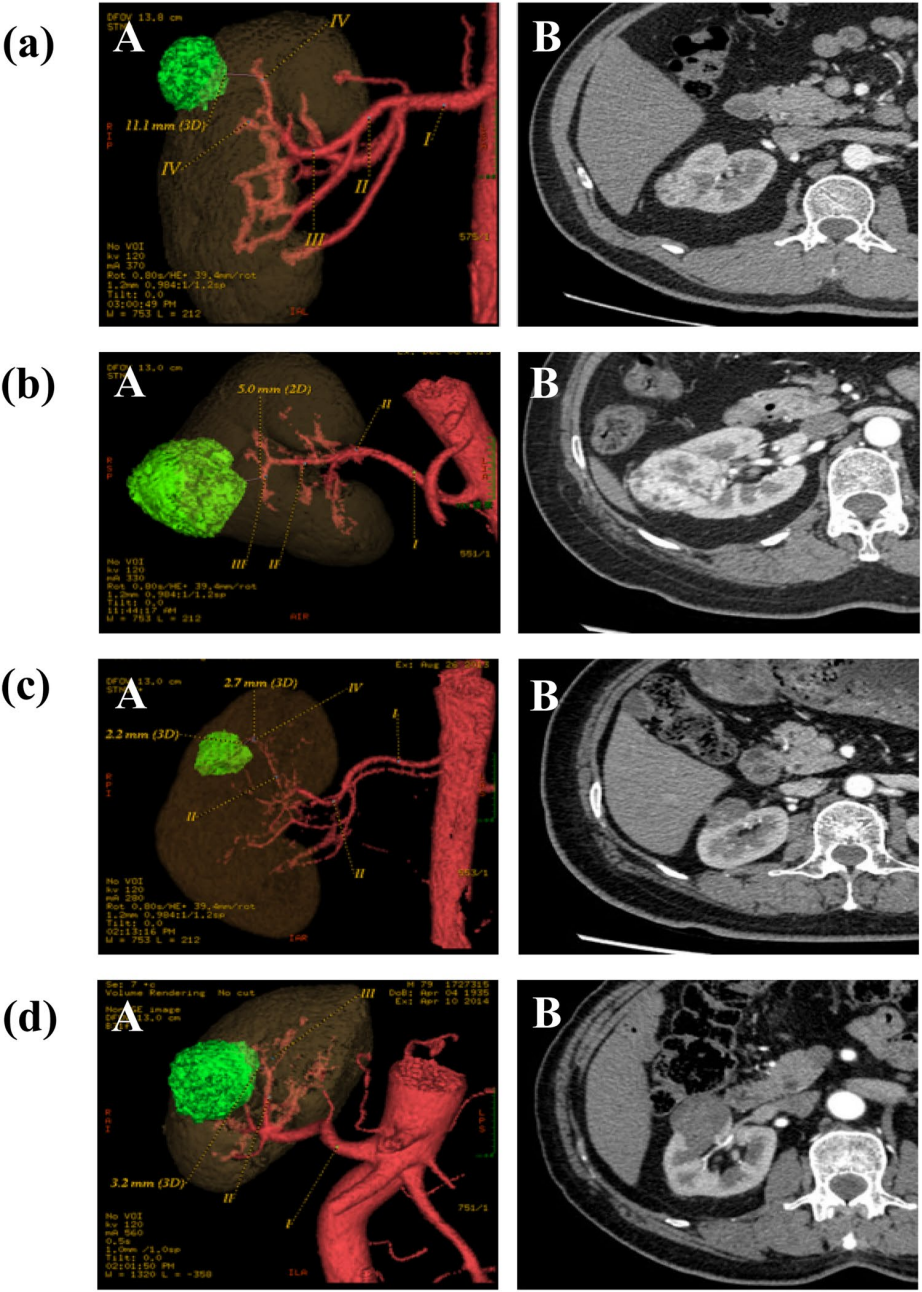
(**a**) 48 year old male patient with right renal mass classified as Peritumoral Artery Scoring System (PASS)-Low risk; (A) VR image; (B) axial image; PAC was N/A. (**b**) 60 year old female patient with right renal mass classified as PASS-Moderate risk: (A) VR image; (B) axial image; PAC was III. (**c**) 61 year old female patient with right renal mass classified as PASS-Moderate risk: (A) VR image; (B) axial image; PAC was IV and PAD ≥2 mm. (**d**) 61 year old male patient with right renal mass classified as PASS-High risk: (A) VR image; (B) axial image; PAC was II.

The inter- and intra-observer data showed excellent concordance with an intraclass correlation coefficients (ICC) >0.8 for all PASS assignment (Fig. 1). Tumors were stratified into the PASS-Low risk group (n = 90), PASS-Moderate risk group (n = 47), and PASS-High risk group (n = 45) (Fig. 2). Baseline patient demographics were similar between groups. Tumors in the PASS-Moderate risk group and PASS-High risk group were larger (p < 0.001), more complex (higher RENAL and nephrometry PADUA score; all p < 0.05), and had larger resected and ischemic volumes than those in the PASS-Low risk group. Warm ischemia time (WIT) of tumors in the PASS-Moderate risk group and PASS-High risk group was longer than that in the PASS-Low risk group (p = 0.009). While no significant difference was found between PASS-Moderate risk group and PASS-High risk group. For renal function outcomes, patients in the PASS-Moderate risk group and PASS-High risk group suffered greater eGFR decline (in terms of both absolute value and as a percentage; all p < 0.001) than those in the PASS-Low risk group at 12-month follow-up.

**Figure 3:**
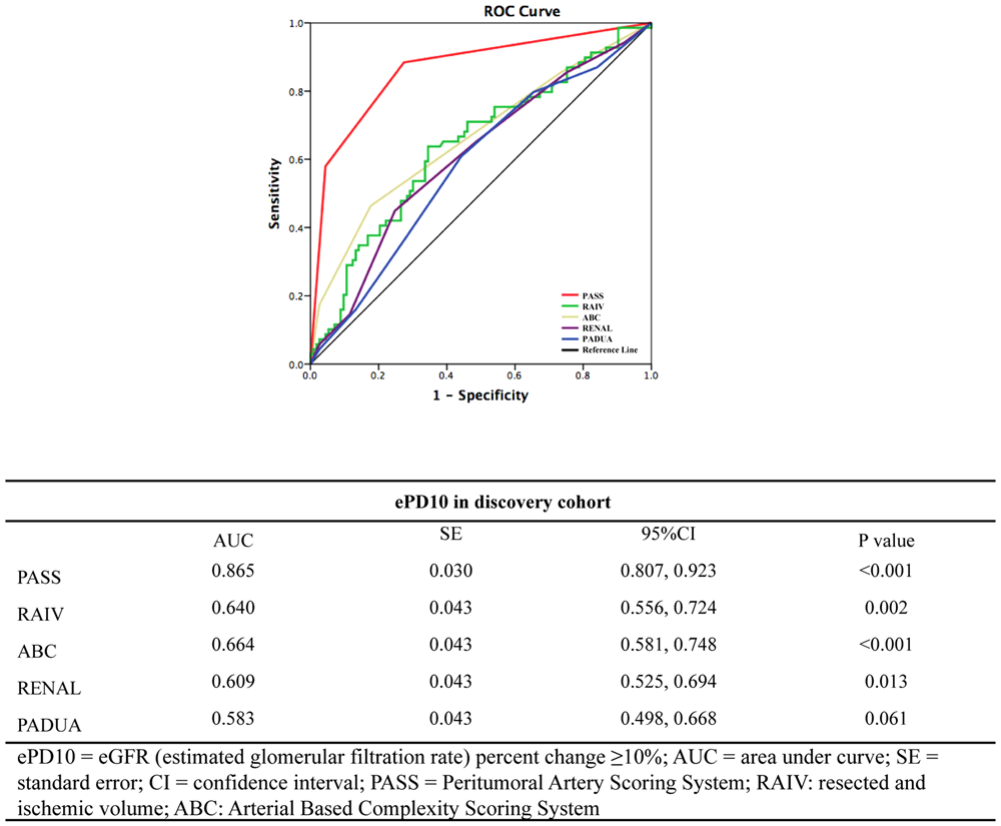
ROC curves for the prediction value of ePD10 between PASS and 4 nephrometry scoring systems in discovery cohort.

Multivariable logistic regression revealed that PASS was significantly superior in predicting ePD10 compared with other perioperative conditions (Table 3). A higher risk category assignment was significantly associated with ePD10. The receiver operating characteristic (ROC) curve analyses of the predictive value of the five nephrometry systems for ePD10 are shown in Fig. 3. PASS was a superior independent predictor of ePD10 for short and long-term outcomes compared with the other systems, and there were considerable differences between the systems (Fig. 3).

**Table 3:** Univariable and multivariable logistic regression for perioperative factors associated with postoperative ePD10 in discovery cohort.

	Univariable analysis		
	OR	95%CI	P value
Age	1.027	1.000, 1.055	0.054
Hypertension	1.064	0.578, 1.959	0.842
Diabetes	1.054	0.420, 2.648	0.910
WIT (per min increase)	1.027	0.955, 1.104	0.474
Malignant vs benign histology	1.296	0.401, 4.188	0.665
Pathological tumor size	1.173	0.945, 1.457	0.147
PASS			
M vs L	5.013	2.199, 11.428	**<0.001**
H vs L	35.744	12.608, 101.337	**<0.001**
RAIV	1.109	1.008, 1.031	**0.001**
ABC			
2 vs 1	0.772	0.343, 1.739	0.533
3S vs 1	1.206	0.460, 3.165	0.703
3H vs 1	6.333	1.523, 26.341	**0.011**
RENAL	1.212	1.008, 1.458	**0.041**
PADUA	1.138	0.957, 1.354	0.145
Age	1.043	1.006, 1.082	**0.022**
Hypertension	1.311	0.593, 2.900	0.504
Diabetes	1.179	0.369, 3.766	0.781
WIT (per min increase)	0.967	0.875, 1.070	0.519
Pathological tumor size	0.999	0.731, 1.367	0.999
PASS			
M vs L	5.604	2.301, 13.649	**<0.001**
H vs L	48.332	14.995, 155.783	**<0.001**
Age	1.037	1.010, 1.064	**0.007**
Hypertension	1.125	0.615, 2.056	0.703
Diabetes	1.055	0.417, 2.668	0.910
WIT (per min increase)	0.982	0.910, 1.059	0.635
Pathological tumor size	0.954	0.706, 1.289	0.758
RAIV (per 20 cm^3^)	1.025	1.007, 1.043	**0.005**
Age	1.039	1.008, 1.070	**0.013**
Hypertension	1.002	0.519, 1.935	0.996
Diabetes	1.064	0.405, 2.796	0.900
WIT (per min increase)	0.980	0.900, 1.067	0.641
Pathological tumor size	1.340	1.021, 1.760	**0.035**
ABC			
2 vs 1	0.573	0.234, 1.400	0.222
3S vs 1	0.792	0.266, 2.352	0.674
3H vs 1	5.450	1.228, 24.179	**0.026**
Age	1.034	1.005, 1.064	**0.022**
Hypertension	1.028	0.544, 1.944	0.931
Diabetes	1.173	0.451, 3.053	0.744
WIT (per min increase)	0.971	0.886, 1.063	0.521
Pathological tumor size	1.201	0.938, 1.538	0.146
RENAL	1.220	0.966, 1.541	0.094
Age	1.034	1.005, 1.064	**0.021**
Hypertension	1.019	0.543, 1.912	0.954
Diabetes	1.216	0.461, 3.207	0.693
WIT (per min increase)	0.992	0.910, 1.082	0.860
Pathological tumor size	1.217	0.949, 1.562	0.122
PADUA	1.103	0.893, 1.362	0.363

**Figure 4:**
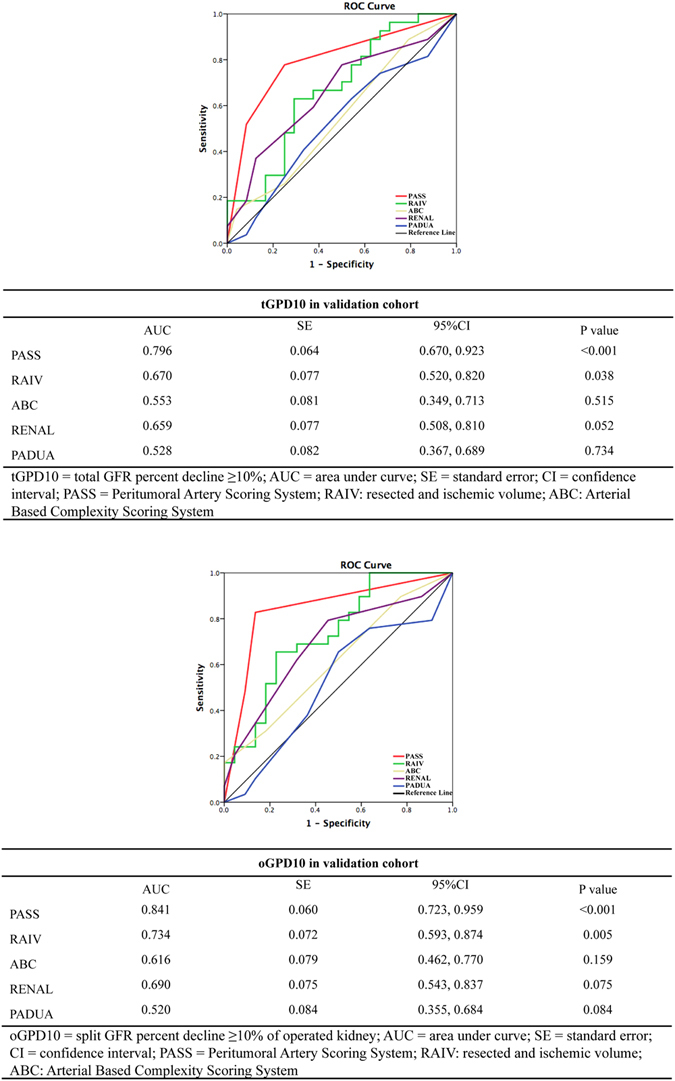
ROC curves for the prediction value of GFR decline in split and total renal function between PASS and 4 nephrometry scoring systems in validation cohort.

Results were validated in an independent cohort (Table 4) and showed superior prediction power of PASS in both split and total postoperative GFR decline (Fig. 4).

**Table 4:** Univariable and multivariable logistic regression for perioperative factors associated with postoperative tGPD10 and oGPD20 in validation cohort.

	tGPD10			oGPD20		
		Univariable analysis			Univariable analysis	
	OR	95%CI	P value	OR	95%CI	P value
Age	1.034	0.984, 1.086	0.181	1.049	0.996, 1.104	0.068
Hypertension	1.187	0.363, 3.881	0.776	1.037	0.314, 3.340	0.952
Diabetes	2.364	0.201, 27.863	0.494	1.556	0.132, 18.340	0.726
WIT (per min increase)	1.198	1.032, 1.391	**0.018**	1.198	1.032, 1.391	**0.018**
Malignant vs benign histology	1.368	0.238, 7.537	0.719	1.913	0.318, 11.518	0.479
Pathological tumor size	1.011	0.642, 1.592	0.963	0.982	0.622, 1.552	0.938
PASS						
M vs L	5.250	1.129, 24.419	**0.034**	38.000	3.889, 371.325	**0.002**
H vs L	21.000	3.664, 120.372	**0.001**	26.600	4.489 157.670	**<0.001**
RAIV	1.028	0.997, 1.059	0.074	1.052	1.006, 1.100	**0.025**
ABC						
2 vs 1	2.179	0.439, 10.830	0.341	2.179	0.439, 10.830	0.341
3S vs 1	1.000	0.132, 7.570	1.000	1.667	0.222, 12.221	1.000
3H vs 1	6.667	0.487, 91.331	0.155	3.667	0.287, 41.331	0.799
RENAL	1.345	0.985, 1.837	0.062	1.444	1.042, 2.001	0.027
PADUA	1.039	0.756, 1.428	0.812	1.030	0.748, 1.418	0.858
	Multivariable analysis			Multivariable analysis		
	OR	95%CI	P value	OR	95%CI	P value
Age	1.004	0.944, 1.069	0.888	1.014	0.942, 1.092	0.707
Hypertension	1.390	0.247, 7.813	0.709	1.013	0.211, 8.501	0.991
Diabetes	19.255	0.334, 110.675	0.153	1.917	0.010, 380.275	0.810
WIT (per min increase)	1.207	1.004, 1.453	0.046	1.293	1.013, 1.649	0.093
Pathological tumor size	0.627	0.300, 1.311	0.215	0.558	0.229, 1.360	0.199
PASS						
M vs L	3.781	1.002, 32.091	**0.049**	28.266	2.277, 355.584	0.010
H vs L	30.116	3.408, 266.148	**0.001**	45.785	3.885, 539.242	**0.002**
	Multivariable analysis			Multivariable analysis		
	OR	95%CI	P value	OR	95%CI	P value
Age	1.024	0.970, 1.080	0.389	1.050	0.982, 1.122	0.152
Hypertension	1.408	0.283, 7.002	0.678	2.466	0.344, 17.680	0.389
Diabetes	3.339	0.143, 78.065	0.453	2.453	0.075, 78.867	0.613
WIT (per min increase)	1.145	0.958, 1.368	0.138	1.166	0.947, 1.436	0.148
Pathological tumor size	0.637	0.314, 1.293	0.212	0.340	0.132, 0.891	**0.028**
RAIV	1.162	0.987, 1.383	0.162	1.112	0.987, 1.213	**0.019**
	Multivariable analysis			Multivariable analysis		
	OR	95%CI	P value	OR	95%CI	P value
Age	1.024	0.966, 1.085	0.426	1.045	0.983, 1.111	0.160
Hypertension	1.240	0.287, 5.387	0.773	1.151	0.252, 5.365	0.869
Diabetes	6.572	0.275, 157.021	0.245	1.324	0.055, 31.617	0.862
WIT (per min increase)	1.227	1.017, 1.480	**0.032**	1.276	1.037, 1.560	0.021
Pathological tumor size	0.938	0.509, 1.727	0.836	0.716	0.411, 1.525	0.485
ABC						
2 vs 1	1.879	0.311, 11.580	0.488	2.676	0.401, 17.580	0.308
3S vs 1	0.840	0.060, 11.795	0.897	3.454	0.208, 57.795	0.388
3H vs 1	1.355	0.053, 34.520	0.854	3.988	0.099, 154.520	0.954
	Multivariable analysis			Multivariable analysis		
	OR	95%CI	P value	OR	95%CI	P value
Age	1.027	0.973, 1.083	0.335	1.041	0.982, 1.103	0.176
Hypertension	1.133	0.256, 5.015	0.870	1.121	0.206, 4.915	0.979
Diabetes	6.191	0.284, 134.763	0.256	1.522	0.075, 31.048	0.785
WIT (per min increase)	1.078	0.990, 1.451	0.064	1.287	1.030, 1.608	0.026
Pathological tumor size	0.903	0.518, 1.575	0.720	0.876	0.466, 1.585	0.618
RENAL	1.077	0.714, 1.626	0.723	1.106	0.719, 1.701	0.648
	Multivariable analysis			Multivariable analysis		
	OR	95%CI	P value	OR	95%CI	P value
Age	1.026	0.971, 1.084	0.360	1.041	0.980, 1.071	0.194
Hypertension	1.434	0.320, 6.318	0.637	1.446	0.270, 7.737	0.667
Diabetes	9.557	0.343, 260.507	0.184	1.883	0.077, 46.153	0.689
WIT (per min increase)	1.316	1.074, 1.612	**0.008**	1.507	1.156, 1.965	**0.002**
Pathological tumor size	0.989	0.566, 1.728	0.969	1.004	0.537, 1.877	0.990
PADUA	0.726	0.466, 1.130	0.156	0.609	0.368, 1.007	0.053

## Discussion

Because multiple small renal masses have low oncological potential, renal functional outcomes of PN contribute significantly to postoperative outcome. Several preoperative and surgical factors have an impact on postoperative renal function after PN, whereas decreasing warm ischemia time (WIT) and minimizing resected parenchymal volume (RPV) are the highest priorities for surgeons who wish to achieve maximal renal function preservation^11^.

In patients with normal preoperative kidney function, who have WIT within acceptable limits, RPV has been suggested as the primary determinant of long-term functional outcome after PN^12^. Recently, several studies showed that having no functional residual renal volume and postoperative renal recovery can also affect postoperative renal functional outcome which was difficult to have precise prediction before surgery^13–15^. Advances in PN techniques have helped to maximize parenchymal volume preservation, leading to the need for a new parameter to predict RPV-unrelated postoperative ePD10^16, 17^.

The renal arterial network consists of the main renal artery, anterior and posterior divisions, and segmental, interlobar, arcuate, and interlobular arteries. Renal artery variants have major clinical implications for the advancement of safe LPN practice^18, 19^. Common variants include the presence of accessory renal arteries, variable points of the renal artery dividing it into the anterior and posterior divisions, and variable origins and courses of segmental renal arteries^17–19^.

Spaliviero *et al*. presented a scoring system based on artery-containing tumors^20^. They showed significant associations between higher category classifications and prolonged ischemia time, more estimated blood loss, and higher urinary fistula risk, but they failed to demonstrate any association with renal function outcome. Despite the small cohort, this study focused on artery-containing tumors, but not the peritumoral arteries, which might be affected during surgery.

The present study defined two major peritumoral artery parameters for evaluation, PAC and PAD, which showed good correlation with long-term renal function outcomes in discovery. Because PAC and PAD were correlated, they were combined in the PASS design. For PAD, 2 mm was a good cut-off value for the prediction of outcomes. MPA was not associated with outcome.

A high or moderate PASS risk category (PAC ≥ III) was significantly associated with renal function decline. To study the mechanism behind this clinical finding, we reviewed the anatomy features of renal artery divisions and here proposed two possible explanations. Firstly, PAC III (segmental artery) reflected a good cut-off value for the prediction of outcomes, because end arteries do not provide adequate collateral circulation^16, 17^. Segmental artery ligation causes irreversible ischemia to the kidney segment and subsequent segmental renal infarction^18^. Surgical injuries and postoperative scar tissue formation could cause hemodynamic changes in the affected artery and decrease effective renal plasma flow, leading to a decrease or loss of function in residual renal volume and cause clinical renal function decline^17, 18, 21^. Secondly, we also observed that tumors of high or moderate PASS risk has significantly higher RENAL nephrometry scores and longer WIT than those of low PASS risk, which indicated PASS risk was associated with tumor complexity. Usually a big, endophytic tumor has more possibility of contact or be close to superior renal artery division^6, 8, 20^. Both high tumor complexity and presence of superior peritumoral artery would increase the difficulty of resection and renorrhaphy procedure under laparoscopy and prolong the WIT, which eventually cause clinical renal function decline^22, 23^.

In the present study, a high PASS risk category was significantly associated with ePD10, GFR percent decline of ≥10% in total renal function (tGPD10) and GFR percent decline of ≥20% in operated renal function (oGPD20). PASS was a superior to other nephrometry systems as an independent predictor of and long-term renal function decline, demonstrating that not only RPV, but also peritumoral artery characteristics affect outcome after LPN.

In discovery cohort, both PASS and Resected and ischemic volume (RAIV)^13^ showed good correlation with ePD10 during multivariable analysis, while only PASS showed great prediction value of tGPD10 in validation. For prediction power comparison, PASS had a higher AUC than RAIV and other 3 nephrometry systems.

Technique repeatability is an important determinant of practical use. Therefore, the evaluation of CTA measurements, such as diameter and artery segmentation, is key in the repeatability of PASS. The PASS utilized volume rendering (VR) to identify the artery segment and Maximum Intensity Projection (MIP) to evaluate the peritumoral-artery relationship because both measurements are easy to perform using a 3D model. Moreover, most imaging stations permit the measurement of VR and MIP. When using 3D CTA for PASS, we recommended that evaluation should be taken in the horizontal and coronal planes. Although strict measurement criteria were not applied, the measurement concordance between the two observers demonstrated the robustness of the PASS.

The present study had limitations because of its retrospective, single study nature. We have registered a prospective, observational, multi-institutional cohort study (ChiCTR-DDD-17010889) to validate the utility of the PASS and improve its accuracy in clinical practice.

## Conclusions

Peritumoral artery characteristics were an independent predictive factor of long-term renal function outcomes after LPN. The PASS based on 3D CTA showed a superior capacity to predict long-term major renal function decline and recovery compared to other nephrometry systems. This study highlights aspects to consider when planning surgical strategies to improve patient safety and long-term clinical outcome.

## Methods and Materials

### Patients and renal function assessment

After approved by board of Renji Renal Cell Carcinoma Database (RRCCD), all patients received LPN-treatment in our hospital between 2013 and 2015 was reviewed retrospectively. Patient demographics, tumor characteristics, WIT, pathological findings, and postoperative functional outcomes were collected and assessed, in accordance with relevant guidelines and regulations and supervised by RRCCD. Patients were informed and had consent signed before been included into RRCCD. The study inclusion criteria were: (1) availability of preoperative available CTA imaging; (2) a single kidney tumor treated in a single surgical session and a normal contralateral kidney; (3) availability of preoperative and postoperative renal function data for at least 12 months. Two-hundred and twenty-two patients treated between 2013 to 2014 were included in discovery cohort. Fifty-one patients, treated in 2015, who underwent perioperative renal scintigraphy during LPN to determine their GFR and have at least 12 months’ follow-up formed our validation cohort.

Renal function was based on serum creatinine (Scr) and eGFR; the latter was based on the modified abbreviated Modification of Diet in Renal Disease equations^24^. Short and long-term postoperative renal function was recorded at approximately three and twelve postoperative months, respectively. For the validation cohort, renal function was evaluated based on split and total GFR measured preoperatively and at the end of the 12-months postoperative follow-up by ^99^Tc^m^ DTPA nuclear renal scintigraphy. Postoperative change in renal function was quantified by the decline in GFR, evaluated for both the operated kidney and for both kidneys combined. An eGFR/GFR percent decline of ≥10% in total renal function or GFR percent decline of ≥20% in operated renal function was considered as major postoperative renal function decline as defined in the ‘trifecta’ outcome^5, 6^.

Surgical specimens were processed using standard pathologic procedures and were evaluated by two experienced pathologists according to the American Joint Committee TNM classification. Surgical margin status was reported.

### Computed tomography arteriography

All CTA examinations were performed using a 64-multidetector computed tomography scanner (VCT LightSpeed, GE Healthcare, Pittsburgh, USA). Patients drank 1000 ml of water before the examination. Four phase images were obtained in a craniocaudal orientation. The unenhanced and arterial, portal, and nephrographic excretory phases spanned the kidneys, the area from the diaphragm to the lower kidney poles, and the kidneys to the symphysis pubis, respectively. Contrast-enhanced images were obtained after intravenous administration of 150 ml of non-ionic contrast medium (Iopamiro, Bracco, Milan, Italy). The scanning parameters of each phase were 110–380 mA, 1.25-mm, and 1.375 of tube current, using current modulation software, collimation, and pitch, respectively. Unenhanced nephrographic and excretory phase scans were reconstructed as 1.25-mm sections. The arterial phase images were reconstructed at 0.725-mm intervals.

### Surgical procedures

Procedures were performed by two senior laparoscopic specialist kidney surgeons. En-bloc hilar clamping to the main renal artery was maintained during tumor excision and renal reconstruction. The renal capsule was incised 3–5 mm from the tumor edge after clamping, and an incision was made between the pseudocapsule and normal renal parenchyma. Subsequently, the tumor was separated from the surrounding parenchyma following the natural plane. The tumor was enucleated along the pseudocapsule during closure of the bottom or approaching the renal sinus. Conventional postresection renorrhaphy was performed. On histopathological review, the mean parenchymal width was 5 mm.

### Peritumoral artery categorization on CTA and PASS design concept

Peritumoral arteries were defined as the renal arteries within 5 mm of the tumor edge, based on the mean parenchymal width. The peritumoral artery was categorized according to the anatomic renal artery division into six classes: peritumoral artery classification (PAC) I, renal artery (arteries, if accessory renal arteries exist); II, anterior and posterior divisions; III, segmental arteries; IV, interlobar arteries; V, interlobar artery branch and arcuate arteries; and VI, no visible peritumoral artery (N/A). The peritumoral artery diameter (PAD) was also measured. If multiple peritumoral arteries (MPA) existed, the peritumoral artery with the higher classification was used for PAC and PAD. CTA images were reviewed by a urologist and a radiologist who specialize in urologic radiology. The inter-observer agreement was calculated. All readouts were performed using an imaging workstation. The individual use of windowing, multiplanar reformations, maximum intensity projection reformats, and VR was allowed, and PAD was measured by 3D VR. PAC was also determined using the VR method. PAD was noted as the average of two measurements. Minimal PAD was limited to 0.5 mm for better inter-measurement repeatability.

PASS was designed to offer a simple and maneuverable anatomically based system for the analysis of PAC and PAD, and to distinguish the tumor subgroups with differing risks for ePD10 after LPN (Fig. 1). PASS-Low risk was defined as PAC IV, V, or N/A and PAD < 2 mm. PASS-Moderate risk was defined as PAC III or PAD ≥ 2 mm. PASS-High risk was defined as PAC I or II with any PAD.

### Statistical Analyses

Analyses were conducted using SPSS^®^ version 21 (IBM Corp., NY, USA). Patient and tumor characteristics were examined using Pearson chi-square and Fisher’s exact tests. Interobserver agreements were assessed using the method proposed by using ICC. ICC values 0–0.20, 0.21–0.40, 0.41–0.60, 0.61–0.80, and 0.81–1.00 reflected slight, fair, moderate, substantial, and excellent agreement, respectively. Univariable logistic regression analysis was used to assess the relationships between renal function outcome and peritumoral artery parameters. Multivariable binary logistic regression analysis was used to evaluate PASS-based prediction of renal function decline when adjusted for perioperative variables (age, hypertension, diabetes, WIT, and tumor size). Five nephrometry systems were included into ROC analysis: PASS, RAIV^13^, Arterial based complexity (ABC) scoring system^20^, RENAL^6^, and PADUA^8^. The predictive values of these five nephrometry systems were compared using ROC and area-under-the-curve (AUC) analysis by calculation with a nonparametric distribution assumption for ePD10, tGPD10 and oGPD20 in the discovery cohort and validation cohort. A P value < 0.05 was considered as statistically significant.
